# Meta-analysis of MitraClip and PASCAL for transcatheter mitral edge-to-edge repair

**DOI:** 10.1186/s13019-024-03218-4

**Published:** 2025-01-03

**Authors:** Mahmoud Balata, Mohamed Ibrahim Gbreel, Mohamed Hamouda Elkasaby, Marwa Hassan, Marc Ulrich Becher

**Affiliations:** 1 Friedrich-Alexander-Universität Erlangen-Nürnberg, Nürnberg, Germany; 2 Faculty of Medicine, October 6 University, Giza Egypt; 3 Faculty of Medicine, Al-Azhar University, Cairo, Egypt; 4 Department of Immunology, Theodor Bilharz Research institute, Giza, Egypt; 5 Department of Internal Medicine II, Städtisches Klinikum Solingen, Solingen, Germany

**Keywords:** MitraClip, PASCAL, Mitral Regurgitation, Transcatheter edge-to-edge repair, Review, And Meta-analysis

## Abstract

**Background:**

Despite the promising results of both MitraClip and PASCAL systems for the treatment of mitral regurgitation (MR), there is limited data on the comparison of both systems regarding their safety and efficacy. We aim to compare both systems for MR.

**Materials and methods:**

Five databases were searched until October 2024. Original studies were only included and critically appraised using an adapted version of the Newcastle–Ottawa scale for observational cohort studies and the Cochrane risk of bias tool for randomized controlled trials. The risk ratio (RR) and mean difference (MD) with their corresponding 95% confidence interval (95% CI).

**Results:**

From the database search, we identified 197 studies, of which eight studies comprising 1,612 patients who underwent transcatheter edge-to-edge repair with either MitraClip or PASCAL were included in this meta-analysis. The statistical analysis revealed no significant difference between the two devices in achieving a two-grade reduction in MR severity (RR = 0.95; 95% CI: [0.86, 1.04]; p = 0.28), one-grade reduction (RR = 1.17; 95% CI: [0.92, 1.49]; p = 0.19), or in cases with no improvement (RR = 1.23; 95% CI: [0.79, 1.90]; p = 0.36). Additionally, there were no significant differences between PASCAL and MitraClip regarding procedure time, procedural success, reinterventions, or all-cause mortality. However, PASCAL trended towards better residual MR reduction, although this was accompanied by moderate heterogeneity. Both devices demonstrated comparable safety profiles and were effective in reducing MR and improving cardiac function.

**Conclusion:**

MitraClip and PASCAL devices showed comparable safety profiles and procedural success rates. However, the analysis did not reveal a statistically significant difference between the two devices in reducing the severity of MR.

**Supplementary Information:**

The online version contains supplementary material available at 10.1186/s13019-024-03218-4.

## Introduction

Valvular heart diseases are a major cause of mortality and reduced quality of life worldwide [1]. One of the most common valvular heart diseases is mitral regurgitation (MR), affecting approximately 1.7% of the US population [2, 3]. Over the past decade, transcatheter edge-to-edge repair (TEER) has emerged as a less invasive alternative to traditional mitral valve repair surgery for high-risk patients suffering from MR. This procedure involves percutaneously approximating the leaflets to restore valvular competence, mimicking the Alfieri stitch used in mitral valve surgery [4–7].

The MitraClip system (Abbott Vascular, Santa Clara, CA, USA) is the leading TEER technique globally. It offers four size variations and an independent grasping feature, making it highly versatile and widely utilized. Another TEER device is the PASCAL system (Edwards Lifesciences, Irvine, CA, USA), which utilizes a Nitinol frame design. This frame is composed of Nitinol, a nickel-titanium alloy known for its shape memory and super-elastic properties including a central spacer designed to fill the regurgitant orifice and prevent backflow from the ventricle to the left atrium, as well as a pair of curved paddles to minimize tension on the grasped leaflets [8]. Despite the proven safety of both systems in the treatment of, there is limited data on direct comparison of the efficacy of the two technologies [9, 10]. Moreover, the risk of mitral valve stenosis might be different between the systems, given the aforementioned difference in the device components [11]. Achieving optimal procedural outcomes, which is MR reduction without creating mitral valve stenosis, is critical to improve patient’s outcomes in the treatment of MR [12].

In this context, we aimed to conduct a meta-analysis to explore the efficacy of the MitraClip and PASCAL systems in terms of the reduction in MR and mitral valve stenosis after the procedure in patients with MR.

## Methods

We conducted the present study based on the Cochrane Handbook of systematic reviews on Interventions [13]. During the process of drafting our manuscript, we strictly followed the recommended reporting items for the Preferred Reporting Items for Systematic Reviews and Meta-Analyses (PRISMA) statement guidelines [14]. Also, the results were reported in line with AMSTAR-2 (Assessing the methodological quality of systematic reviews 2) guidelines [15].

### Protocol registration

The protocol of this systematic review followed the recent update of PRISMA statement [14] guidelines and was upfront registered in the PROSPERO registry (CRD42023410540).

### Search strategy

The following electronic databases were systematically searched: PubMed, Web of Science (WOS), Scopus, Cochrane library, and Medline via Ovid until October 2023. We used the following search strategy: (Mitral) AND (Insufficiency OR Incompetence OR Regurgitation) AND (MitraClip) AND (Pascal). All the included studies' references were screened to avoid missing any studies and guarantee high-quality screening *(*Additional file 1: Table S1 *for the detailed strategy of each database).*

### Eligibility criteria

In this meta-analysis, we focused on studies that included patients diagnosed with mitral regurgitation and who underwent TEER using either the MitraClip or PASCAL devices. We considered both randomized and non-randomized clinical trials (RCTs), as well as prospective or retrospective observational studies. We excluded non-human studies and other types of study designs, including conference abstracts, cross-sectional studies, case reports, case series, and studies that were not written in English. By implementing these strict inclusion and exclusion criteria, we aimed to ensure that the studies we analyzed were highly relevant to our research question and provided the highest quality evidence possible to inform our meta-analysis (Additional file 1).

### Screening and study selection

Using Endnote software, we collected the different records from the different databases and removed duplicates [16]. The retrieved references were screened to assess their relevance. The screening was done in two steps; title and abstract screening, followed by full-text screening for final eligibility. Each step was done at least by two independent authors, and the findings were compared, and group discussions then solved disagreements.

### Quality assessment

For all RCTs that were included, the Cochrane Collaboration tool version two was used to evaluate their quality [17]. It encompasses the following domains: randomization process, Deviations from intended interventions, Missing outcome data, Measurement of the outcome, Selection of the reported result, and other sources of bias (Additional files 2 & 3). The evaluation is based on a determination of whether there is a low, high, or unclear bias risk. Cohort studies were assessed using the Newcastle Ottawa scale [18].

### Data extraction

The data was extracted from the included studies manually via utilizing Microsoft Excel worksheet [19]. Extracting summary of data included the study ID (last name of the first author et al.), year of publication, the study setting and design, follow-up duration, sample size, population definition, primary outcomes, MR quantifying assessment tool, and the generation of MitraClip or PASCAL used.

Baseline characteristics of the enrolled participants included study arms, the number of sample size, age, gender, body mass index, Society of Thoracic Surgeons score for mitral valve repair, Euro-SCORE II, New York Heart Association (NYHA) functional class, comorbidities (atrial fibrillation, diabetes mellitus, hypertension, myocardial infarction, stroke), and echocardiographic measures (etiologies of MR, the severity of MR, mitral valve area, effective regurgitant orifice area, left ventricular ejection fraction, transmitral mean gradient, and pulmonary artery systolic pressure). Disagreements were solved later by group discussion.

### Primary and secondary endpoints

The primary endpoint was a reduction in MR. The secondary endpoints included reductions in vena contracta width and effective regurgitant orifice area (EROA), mean mitral gradient at discharge, procedural success, device success, major bleeding, reintervention, and all-cause mortality after TEER.

### Statistical analysis

We used R version 4.2.2 (2022–10–31) and R Studio version 2022.07.2 (2009–2022) RStudio, Inc.). Dichotomous data were analyzed as risk ratio (RR) and 95% confidence interval (CI) and continuous data as mean difference (MD) and 95% CI. Statistical heterogeneity among the studies was assessed by visual inspection of the forest plot, besides using I-squared (I2.) and chi-squared (Chi2) statistics. I^2^ values of 50% were indicative of high heterogeneity. A random-effects model was applied when there was a significant variation in the data (P < 0.1, and I2 > 50%). Other than that, the fixed-effect model was applied. We resolved the heterogeneity by leaving one out meta-analysis.

## Results

### Literature search and study selection

**Fig. 1:**
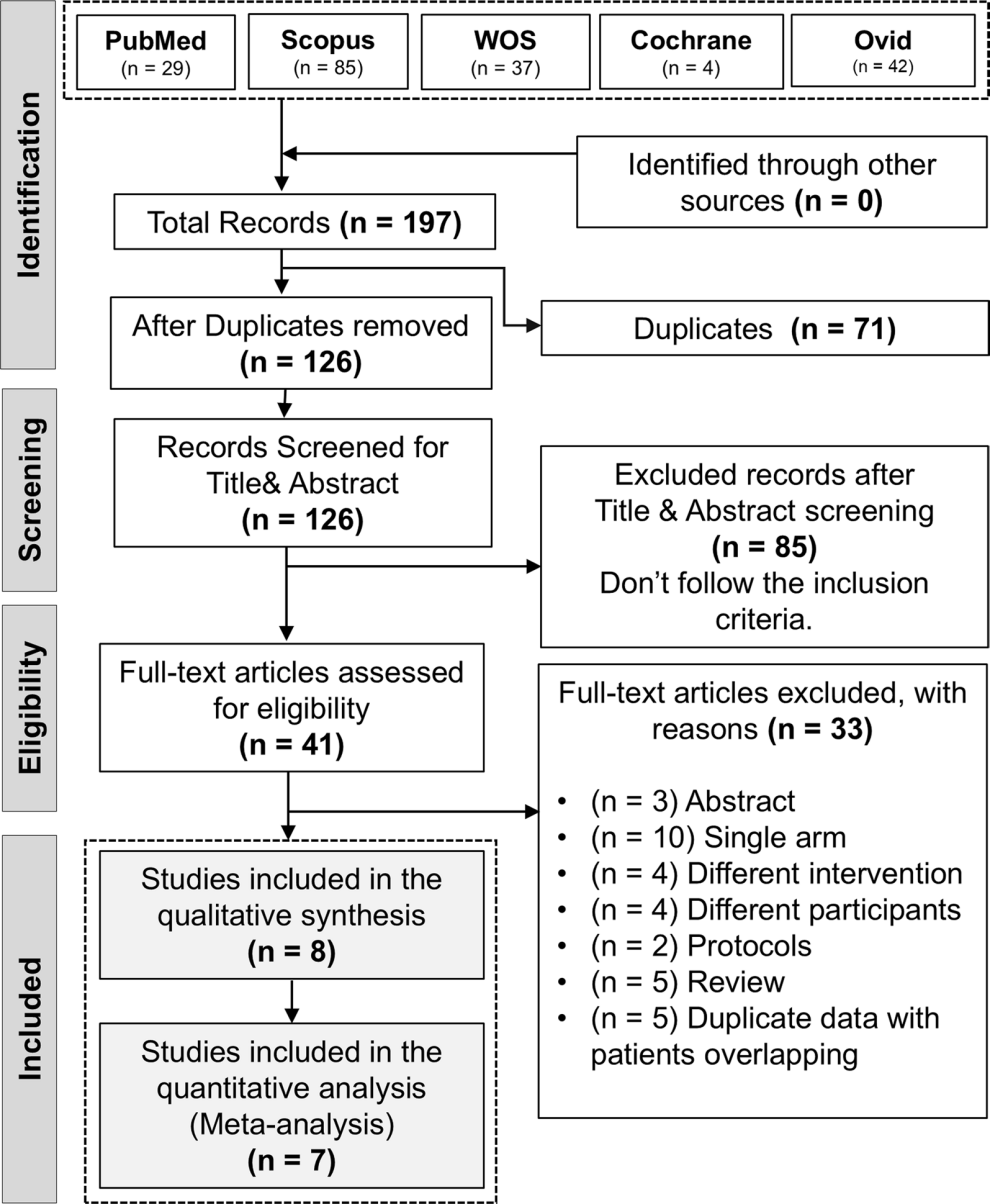
PRISMA flow diagram of the literature search results

The computerized search and reference lists of the databases yielded 197 articles. After removing duplicates, 126 articles were screened. Following the primary screening, 41 articles were included. The full texts of these papers were processed for secondary screening; eight articles [10, 11, 20–25] were included in the qualitative synthesis of the study, and seven of them [10, 11, 20–24] were included in the meta-analysis. (Fig. 1*.* PRISMA flow diagram of the literature search results).

### Characteristics of the included studies

Table 1 provides an overview of the studies that were included. All the included studies were cohort studies [11, 20–24], except one was a randomized controlled trial [10]. A total of 1612 patients were investigated. Tables 2, 3 shows the detailed characteristics of the participants in the included studies.

**Table 1:** Summary of the included studies

Study ID	Year	Setting	Design	Sample Size	Follow-up duration	Population definition	Primary outcome measures	Study Duration	MR quantifying assessment tool	MitraClip generation	PASCAL generation
Barth et al. [25]	2021	Germany	Cohort	31	5 months	Patients with symptomatic severe MR was quantified by echocardiography and cardiac catheterization in accordance with the guidelines on valvular heart disease of the European ESC	Successful implantation Hemodynamic and echocardiographic results	Between July 2019 and February 2020	Echocardiography and cardiac catheterization	N/R	Edwards Lifescience, Irvine, CA, USA
Geis et al. [21]	2022	Germany	Cohort	123	12 months	All patients undergoing primary TEER with third generation MitraClip (NTR/XTR) from March 2018 to March 2020 as well as the first patients implanted with a PASCAL device until March 2020	Device success and procedural success	Between March 2018 and March 2020	Echocardiography and cardiac catheterization	Third generation MitraClip (NTR/ XTR)	Edwards Lifescience, Irvine, CA, USA
Gerçek et al. [22]	2021	Germany	Cohort	38	12 months	Patients with complex PMR (defined as mitral regurgitation effective regurgitant orifice area (MR-EROA) ≥ 0.40 cm2 or large flail gap (≥ 5 mm) or width (≥ 7 mm) or Barlow`s disease)	reduction at least to moderate MR (2 +) at the end of the procedure, successful access and retrieval of the device delivery system, and procedural success	Between August 2018 and April 2020	Echocardiography	Third generation MitraClip (NTR/ XTR)	Edwards Lifescience, Irvine, CA, USA
Lim et al. [10]	2022	Germany	Cohort	180	6 months	Age ≥ 18 years, grade 3 + or 4 + DMR by TTE or TEE as assessed by the echocardiography core laboratory	The proportion of patients with MR ≤ 2 + at 6 months as assessed by the echocardiography core laboratory	Between November 2018 and December 2021	Echocardiography	Third generation MitraClip (NTR/ XTR)	Edwards Lifescience, Irvine, CA, USA
Mauri et al. [11]	2022	Germany	Cohort	614	51 days	Patients commercially treated with the PASCAL repair system or MitraClip for symptomatic moderate-to-severe or severe MR	Technical success and severity of MR at discharge on intention-to-treat analysis (all patients with procedure initiated)	Between February 2019 and December 2019	Echocardiography	Third generation MitraClip (NTR/ XTR)	Edwards Lifescience, Irvine, CA, USA
Schneider et al. [23]	2022	Germany	Cohort	412	12 months	Patients who underwent treatment with the MC or PASCAL repair system between April 2018 and December 2020	Technical success and severity of MR at discharge	Between April 2018 and December 2020	Echocardiography and cardiac catheterization	Third generation MitraClip (NTR/ XTR)	Edwards Lifescience, Irvine, CA, USA
Errthum et al. [24]	2021	USA	Patient-specific MV-LV model	N/R	N/R	N/R	N/R	N/R	N/R	Third generation MitraClip (NTR/ XTR)	Edwards Lifescience, Irvine, CA, USA
Haschemi et al. [20]	2022	Germany	Cohort	214	1 month	Patients treated with either the PASCAL or the MitraClip system	Short-term follow-up period was within 30 days after implantation. Residual MR ≤ 1 + , technical success rates,30-day mortality and long-term outcomes were similar in both groups	between June 2019 and August 2021	Local echocardiography	MitraClip NTR/XTR in 90 patients, G4 system in 22 patients	Edwards Lifescience, Irvine, CA, USA

ESC: Society of Cardiology, MR: Mitral Regurgitation, N/R: Not reported, TEER: Transcatheter Edge-to-Edge Repair, MR-EROA: Mitral Regurgitation- effective regurgitant orifice area, PMR: primary mitral regurgitation, TTE: transthoracic echocardiography, TEE: transesophageal echocardiography

**Table 2:** Baseline characteristics of the included studies

Study ID	Arms	Sample	Age (years), mean (SD)	Gender, Male, n (%)	BMI, kg/m2 mean(SD)	STS score for mitral valve repair, % mean(SD)	Euro-SCORE II, % mean(SD)	NYHA functional class n(%)				
								I	II	III	IV	III or IV
Barth et al. [25]	PASCAL and MitraClip Propensity matched	31	77.5 (13.25)	19(61.3)	27.6 (6.2)	9.1 (7.4)	10.2 (14.5)	-	-	-	-	31 (100)
Errthum et al. [24]	PASCAL and MitraClip	-	-	-	-	-	-	-	-	-	-	-
Geis et al. [21]	MitraClip	82	77.5 (18.9)*	45 (54.88)			6.55 (9.8)*	-	-	-	-	72(87.8)
	PASCAL	41	74.4 (18.1)*	24 (58.53)			5.1 (4.8)*	-	-	-	-	36(87.8)
Gerçek et al. [22]	MitraClip	16	81.8(8.1)	9 (56.25)	25 (4.1)	2.367(2.4)	4.2(3)	-	-	-	-	-
	PASCAL	22	81.9(6.2)	13 (59.1)	25.9(3.8)	3.167(2.7)	4.6(3.7)	-	-	-	-	-
Lim et al. [10]	MitraClip	63	81.2 (6.2)	43 (68.3)	26.2 (4.8)	3.6 (2.2)	4.1 (3.1)	-	-	-	-	39(61.9)
	PASCAL	117	81.1 (6.9)	78 (66.7)	25.9 (5.4)	4.1 (2.8)	3.9 (2.9)	-	-	-	-	71(60.7)
Mauri et al. [11]	MitraClip	307	77.1 (8.5)	178(58)	-	-	6.9 (4.9)	-	52 (16.9)	218 (71.0)	37 (12.1)	-
	PASCAL	307	77.0 (9.6)	177 (57.7)	-	-	5.8 (4.5)	-	43 (14.0)	236 (76.9)	28 (9.1)	-
Schneider et al. [23]	MitraClip	216	77 (9)	109 (50.5)	25.6 (4.7)	5.5 (5.5)	7.2 (7.0)	4 (1.9)	27 (12.6)	150 (69.7)	34 (15.8)	-
	PASCAL	196	76 (12)	120 (61.2)	25.9 ( 5.5)	4.3 (3.6)	5.8 (4.9)	2 (1.0)	15 (7.7)	149 (76.0)	30 (15.3)	
Haschemi et al. [20]	MitraClip	112	-	-	-	-	-	-	95 (86)	-	-	-
	PASCAL	102	-	-	-	-	-	-	78 (78)	-	-	-

^*^Data is presented as median (IQR)

**Table 3:** Comorbidities and Echocardiographic findings of the included patients in the included studies

Study ID	Study Arms	Comorbidities n (%)						Echocardiographic measures							
		AF	DM	HTN	MI	Stroke	DMR, n (%)	FMR etiology, n (%)	MR 3 + , n(%)	MR 4 + , n(%)	Mitral valve area, cm2 mean(SD)	Effective regurgitant orifice area, cm2 mean(SD)	LVEF, % mean(SD)	Transmitral mean gradient, mm Hg mean(SD)	Pulmonary artery systolic pressure, mm Hg mean(SD)
Barth et al. [25]	PASCAL and MitraClip Propensity matched	19 (61.3)	9 (29.0)	30 (96.8)	6 (19.4)	3 (9.7)	9 (29.0)	19 (61.3)	31 (100)	-	-	0.38 (0.2)	-	-	-
Errthum et al. [24]	PASCAL and MitraClip	-	-	-	-	-	-	-	-	-	-	-	-	-	-
Geis et al. [21]	MitraClip	57(69.51)	25(30.49)	68(82.92)		12(14.63)	32(39.02)	50(60.98)	-	-	-	-	-	1.11 (IQR 0.84)*	49 (18.25)*
	PASCAL	27(65.85)	13(31.7)	33(80.49)		5(12.2)	14(34.15)	27(65.85)	-	-	-	-	-	1.21 (IQR 0.89)*	53 (18.5)*
Gerçek et al. [22]	MitraClip	10(62.5)	1(6.3)	-	2(12.5)	4(25)	-	-	-	-	4.8(1.9)	0.7(0.3)	-	2.5(0.8)	-
	PASCAL	15(68.2)	4(18.2)	-	1(4.5)	3(13.6)	-	-	-	-	4.6(1.4)	0.74(0.12)	-	2(1.6)	-
Lim et al. [10]	MitraClip	38(60.3)	15(23.8)	57(90.5)	7(11.1)	1(1.6)	63(100)	-	13(20.6)	50(79.4)	-	0.50 (0.20)	58.3 (9.0)	2.4 (1.1)	45.6 (14.6)
	PASCAL	67(57.3)	19(16.2)	98(83.8)	19(16.2)	9(7.7)	117 (100)	-	29(25.2)	86(74.8)	-	0.50 (0.15)	59.6 (8.7)	2.5 (1.1)	42.3 (11.4)
Mauri et al. [11]	MitraClip	195 (63.5)	81 (26.4)	249 (81.1)	92 (30.0)	-	103 (33.6)	204 (66.4)	107 (34.9)	200 (65.1)	-	0.34 (0.13)	47 (15)	2.2 (1.1)	-
	PASCAL	217 (70.7)	76 (24.8)	272 (88.6)	50 (16.3)	-	101 (32.9)	206 (67.1)	121 (39.4)	186 (60.6)	-	0.39 (0.22)	47 (15)	1.9 (1.1)	-
Schneider et al. [23]	MitraClip	123 (56.9)	38 (27.9)	102 (75.0)	28 (14.3)	18 (8.3)	70 (35.2)	93 (46.7)	212 (98.6)	139 (64.6)	3.7 (1.5)	-	45 (15)	1.9 (1.4)	52 (16)
	PASCAL	129 (65.8)	45 (23.0)	166 (84.7)	56 (25.9)	39 (19.9)	80 (40.8)	101 (51.5)	193 (98.5)	92 (46.9)	4.3 (1.6)	-	50 (15)	2.2 (1.2)	44 (16)
Haschemi et al. [20]	MitraClip	-	-	-	-	-	-	-	-	-	-	-	-	-	-
	PASCAL	-	-	-	-	-	-	-	-	-	-	-	-	-	-

AF: Atrial Fibrillation, DM: Diabetes Mellitus, HTN: Hypertension, MI: Myocardial Infarction, DMR: Degenerative mitral regurgitation, FMR: Functional mitral regurgitation

### Quality assessment

**Fig. 2:**
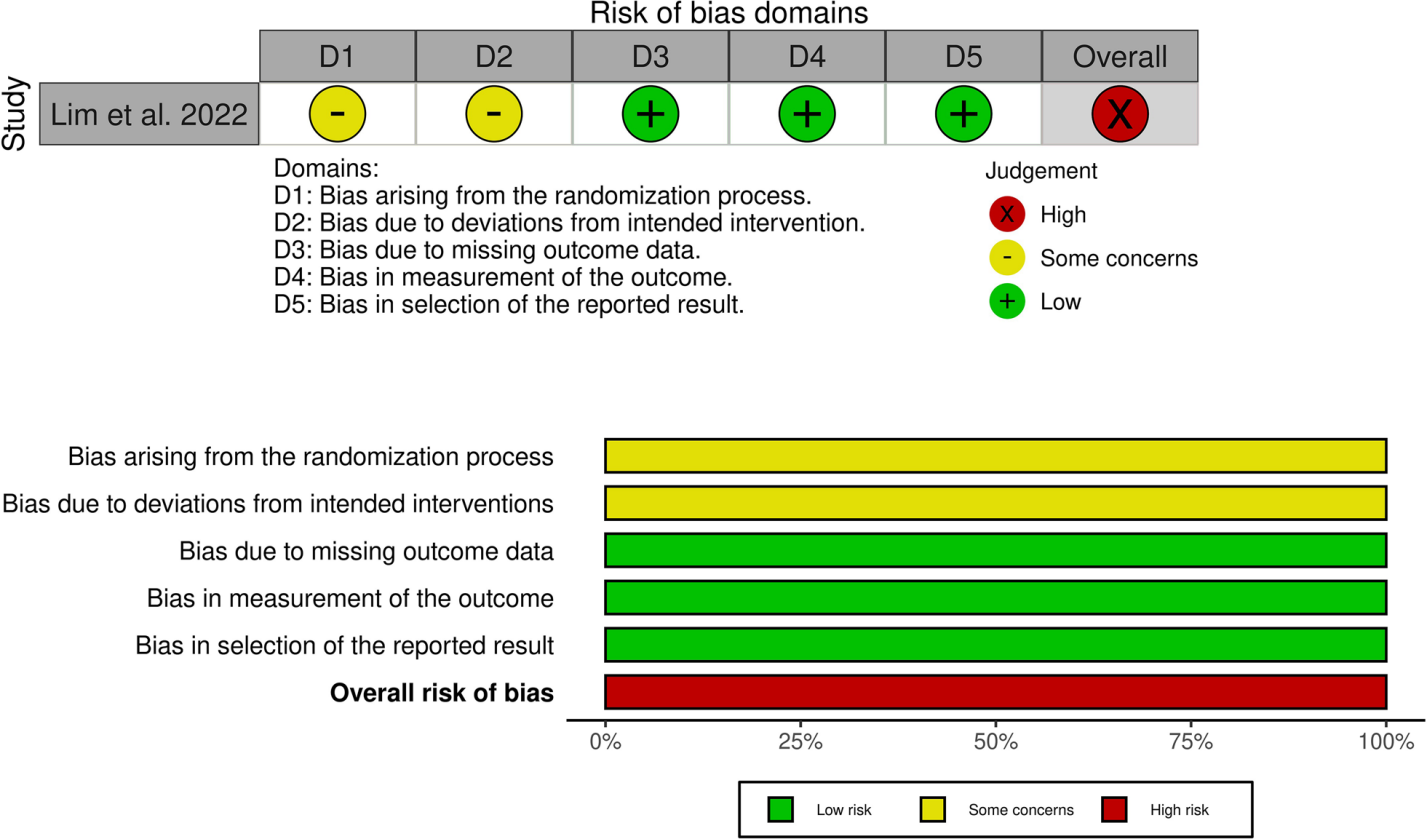
Cochrane risk of bias (ROB) tool version 2 for Lim et al. quality assessment as a randomized controlled trial

According to the Newcastle–Ottawa scale, all included studies were low risk of bias except Errthum et al. 2021 [24]. Detailed information about quality assessment is reported in Additional files 2, 3, respectively. According to the Cochrane tool version two, Lim et al. 2022 [10] study has some concerns in terms of the randomization process and deviations from intended interventions (Fig. 2).

### Outcomes

#### MR reduction at discharge

**Fig. 3:**
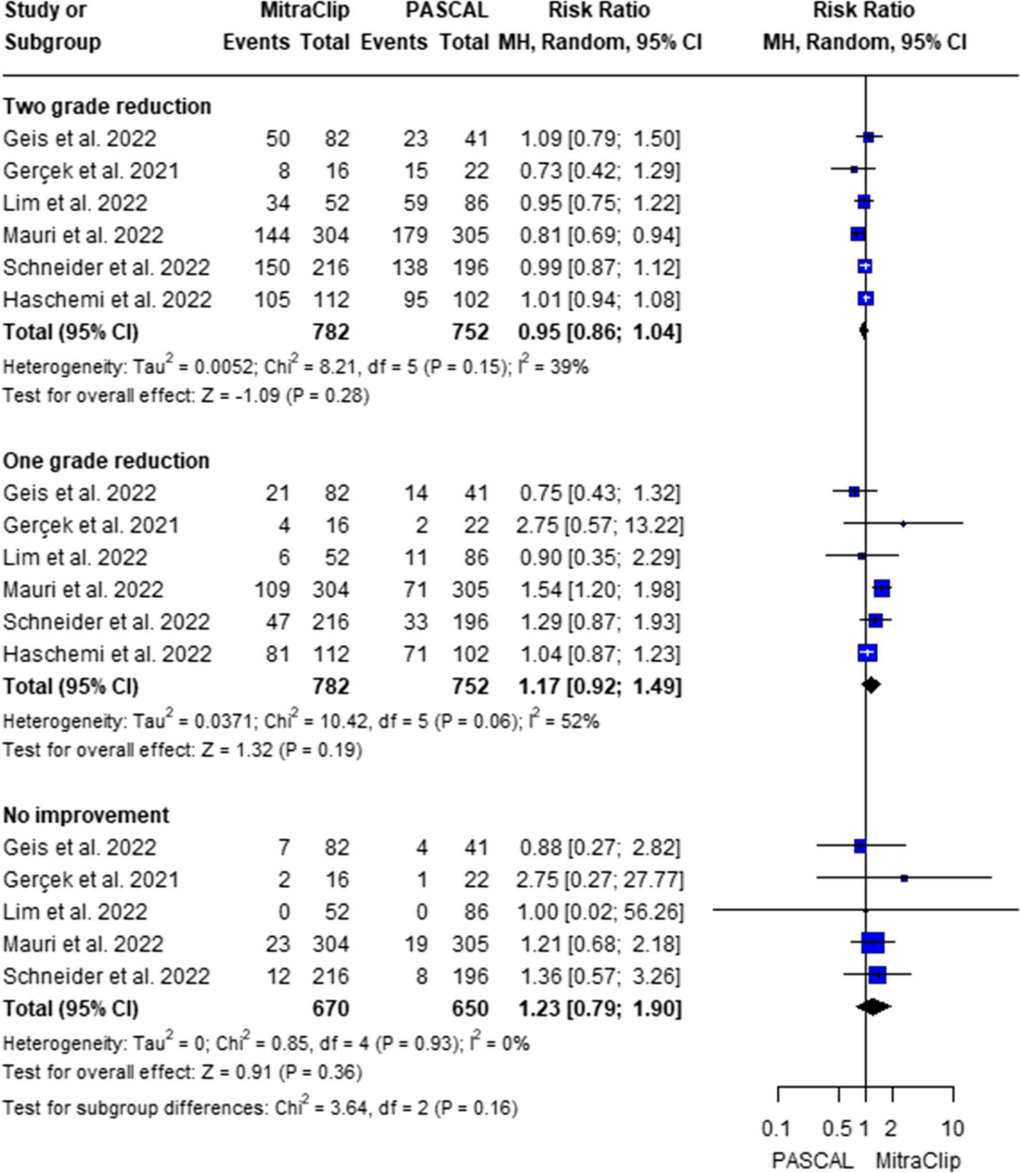
Forest plot of risk ratio (RR) and 95% confidence interval (CI) in the degree of MR declining

The analysis showed no significant difference between the MitraClip and PASCAL systems in achieving a two-grade reduction in mitral regurgitation (RR = 0.95; 95% CI: [0.86, 1.04]; p = 0.28; heterogeneity, p = 0.15; I^2^ = 39%). Similarly, for one-grade reduction, there was no significant difference between the two devices (RR = 1.17; 95% CI: [0.92, 1.49]; p = 0.19; heterogeneity, p = 0.06; I^2^ = 52%). In terms of patients with no improvement, the results also showed no significant difference between MitraClip and PASCAL (RR = 1.23; 95% CI: [0.79, 1.90]; p = 0.36; heterogeneity, p = 0.93; I^2^ = 0%). Therefore, the overall analysis indicated no statistically significant difference between the PASCAL and MitraClip systems in reducing the severity of mitral regurgitation (Fig. 3).

#### Vena contracta width

**Fig. 4:**
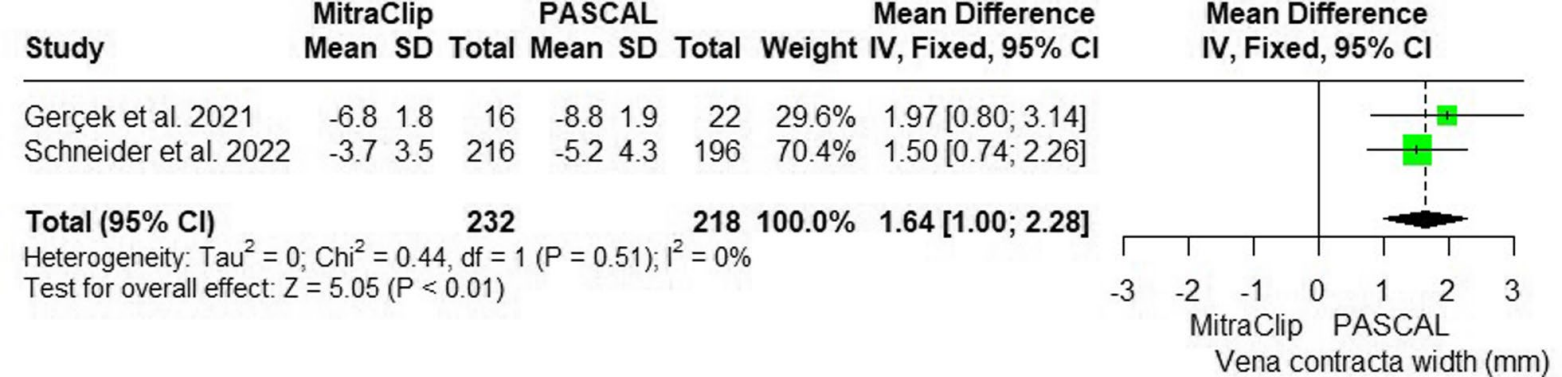
Forest plot of mean difference (MD) and 95% confidence interval (CI) in vena contracta width (mm)

The PASCAL system was more effective than the MitraClip in reducing vena contracta width, with a MD of 1.64 mm (95% CI: [1, 2.28]; p < 0.01). The two pooled studies (21 and 22) showed no heterogeneity (p = 0.51; I^2^ = 0%) (Fig. 4).

#### Effective regurgitant orifice area (EROA)

**Fig. 5:**
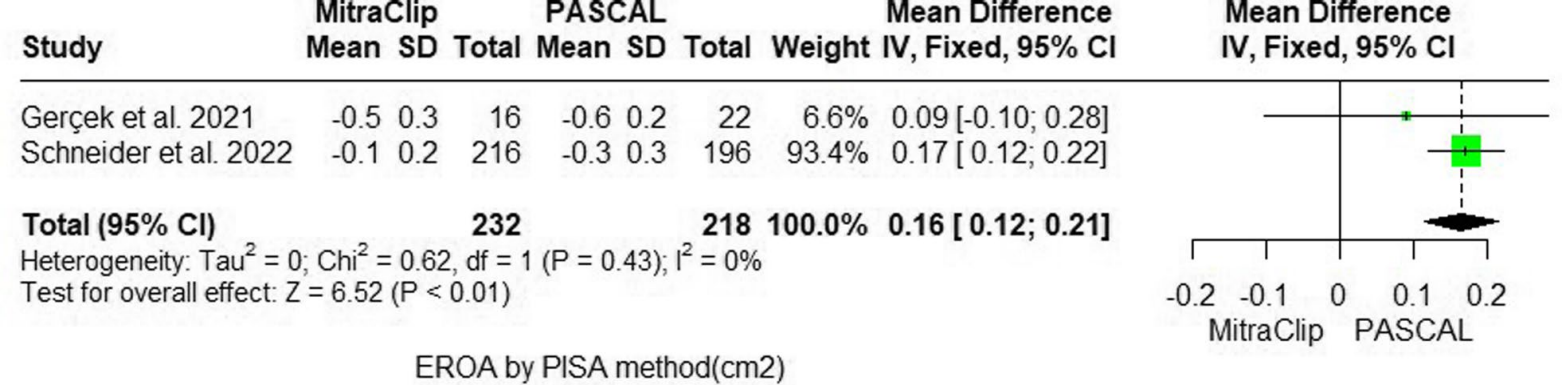
Forest plot of mean difference (MD) and 95% confidence interval (CI) in effective regurgitant orifice area (EROA) (cm2) via proximal isovelocity surface area (PISA) method

The PASCAL system demonstrated significantly better performance in reducing EROA than the MitraClip system (MD = 0.16 mm^2^; 95% CI: [0.12, 0.21]; p < 0.01). The two studies (21 and 22) included in the analysis were homogeneous (P = 0.43; I^2^ = 0%) (Fig. 5).

#### Transmitral mean pressure gradient at discharge

**Fig. 6:**
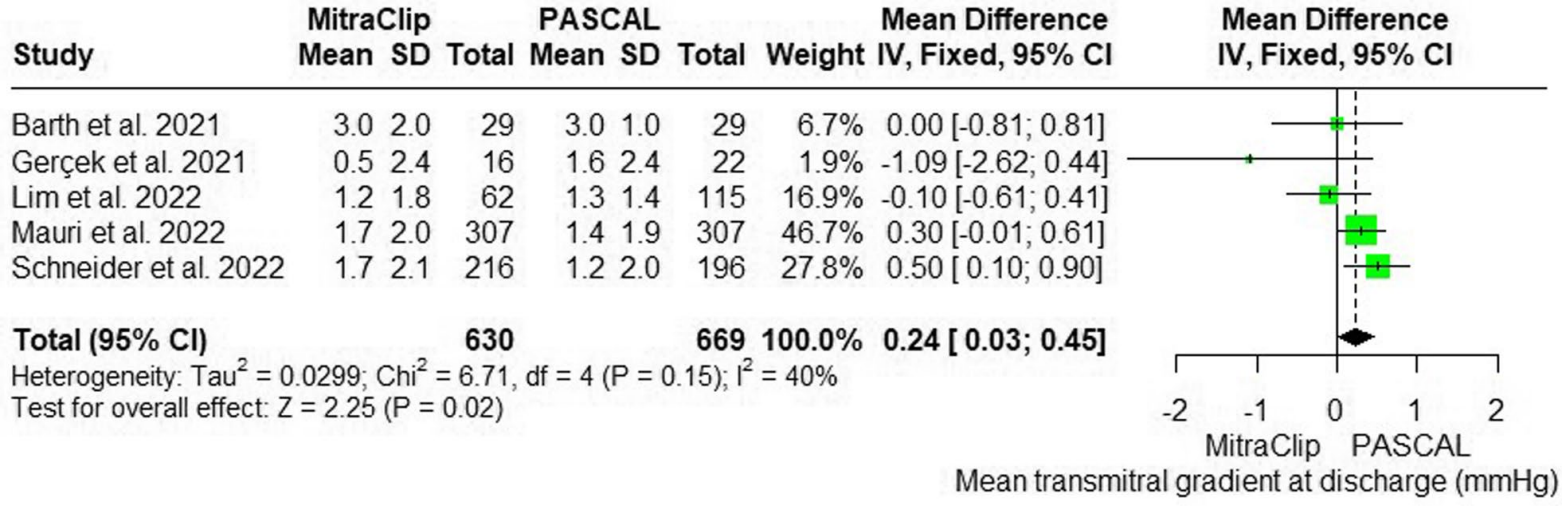
Forest plot of mean difference (MD) and 95% confidence interval (CI) in trans mitral pressure gradient at discharge (mmHg)

Five studies reported the mean transmitral pressure gradient at discharge. The meta-analysis revealed a favorable result of PASCAL over MitraClip, showing that PASCAL was associated with a significantly lower transmitral mean pressure gradient at discharge than MitraClip (MD = 0.24 mmHg; 95% CI: [0.03, 0.45]; p = 0.02). No significant heterogeneity was detected between the pooled studies (p = 0.15; I^2^ = 40%) (Fig. 6).

#### Procedure time

There was no significant difference in procedure time between the MitraClip and PASCAL systems (MD = 4.75; 95% CI: [−15.12, 24.63]; p = 0.64; Heterogeneity: p < 0.01; I^2^ = 75%). A sensitivity analysis (leave-one-out test) resolved the heterogeneity by excluding the study of Barth et al. 2021, and the results remained not significant (MD = −2.56; 95% CI: [−7.10, 1.97]; p = 0.27; Heterogeneity: I^2^ = 0%) (Additional file 4: Fig. S1& S2).

#### Procedural success

Procedural success was defined as a reduction of MR degree to less than moderate (2 +). Our meta-analysis showed no significant difference in procedural success between the MitraClip and PASCAL systems (RR = 1.00; 95% CI: [0.98, 1.02]; p = 0.89; Heterogeneity: p = 0.73; I^2^ = 0%). (Additional file 4: Fig. S3).

#### Device success

Device success was defined as the reduction of mitral regurgitation (MR) by at least one grade, with a final MR grade of ≤ 2 + . Our analysis revealed no significant difference in device success rates between the MitraClip and PASCAL systems (RR = 0.95; 95% CI: [0.90, 1.00]; p = 0.06; Heterogeneity: p = 0.46; I^2^ = 0%) (Additional file 4: Fig. S4).

#### Major bleeding

No significant difference in major bleeding between MitraClip and PASCAL was found in our analysis (RR = 0.59; 95% CI: [0.25, 1.40]; p = 0.23; Heterogeneity: p = 0.48; I^2^ = 0%) (Additional file 4: Fig. S5).

#### Reintervention

There was no significant difference in reintervention rates between the MitraClip and PASCAL devices (RR = 0.67; 95% CI: [0.23, 1.90]; p = 0.45; Heterogeneity: p = 0.91; I^2^ = 0%) (Additional file 4: Fig. S6).

#### All-cause mortality

No significant difference in all-cause mortality was found between the MitraClip and PASCAL systems (RR = 1.73; 95% CI: [0.92, 3.27]; p = 0.09; Heterogeneity: p = 0.82; I^2^ = 0%) (Additional file 4: Fig. S7).

## Discussion

The present study was aimed to conduct a systematic review and meta-analysis comparing two TEER mitral repair systems, MitraClip and PASCAL. The major findings can be summarized as follows: PASCAL demonstrated superior reduction of the MR grade, vena contracta and EROA compared to MitraClip. In addition, PASCAL was found to be associated with a lower transmitral pressure gradient at discharge. Both systems yielded comparable outcomes in terms of procedural success, major bleeding, reintervention, and all-cause mortality. These findings are crucial as reducing the severity of MR can alleviate strain on the heart, improve symptoms, and quality of life, and decrease hospitalization due to heart failure and mortality rates in patients with MR [26–28].

Our findings are consistent with the multicentric propensity score matching analysis of Mauri et al. (2022), which reported that the PASCAL system more often led to MR degree ≤ 1 + compared to the MitraClip system after the procedure (70% vs. 57%, p < 0.001) [11]. Geis et al. (2022) also showed those who received PASCAL showed significantly higher incidence of residual MR ≤ trace at follow-up time points (1–4 months: p = 0.0081; 6–18 months: p = 0.0017) [21]. The unique design of PASCAL, with its multidimensional catheter steering options and independent leaflet capture, may facilitate optimal leaflet insertion with optimal alignment to achieve the better reduction in MR. In addition, the central spacer may play a role in reducing the defect gap and the regurgitant volume. It is important to note that newer generations of MitraClip devices are bigger in size, with longer and broader arms and optional independent leaflet grasping. Further research studies should be conducted to assess the procedural the performance of the newer generations of TEER devices Additional file 5.

Another important finding of our meta-analysis is that PASCAL was associated with a significantly lower transmitral pressure gradient at discharge compared to MitraClip, infering that PASCAL can effectively restore mitral valve competency with less degree of stenosis. This finding holds significant importance, as a higher post-interventional transmitral pressure gradient has been linked to poorer outcomes after TEER in patients with MR [29, 30]. Significant differences in mean pressure gradient between the two repair systems could not be reported by the studies conducted by Gerçek M et al. (2021) [22], Lim DS et al. (2022) [10], and Schneider L et al. (2022) [23]. However, it should be noted that those studies had limitations in their sample size or patient population. For instance, Gerçek M et al. (2021) investigated only 22 patients in the PASCAL group and 16 in the MitraClip group [22].

A recently published work by Hosseini et al. [31] concluding no significant differences between PASCAL and MitraClip groups in short-term all-cause mortality rates, MR reduction to 2 + or less at discharge, or improvement in NYHA class at follow-up. They showed that both devices demonstrated high success rates for TEER, with no difference between them. PASCAL was not inferior to MitraClip in reducing regurgitation to MR ≤ 1 + at discharge, although heterogeneity was high. However, they obtained higher heterogeneity compared to ours. Subgrouping in our meta-analysis enabled us to obtain a high-quality evidence. Adverse events were generally low in incidence in their study.

Similar to our results, another research correspondence conducted by Bansal et al. [32] compared the PASCAL and MitraClip devices for mitral valve repair in a cohort of 1,581 patients. They found no significant differences in procedural success, 30-day mortality, or post-procedure gradients between the two devices. However, they observed a trend toward better residual MR reduction with the PASCAL device, though this result was accompanied by moderate heterogeneity among the included studies. Furthermore, Bansal et al. reported that while both devices were equally safe and associated with low mortality rates, the PASCAL device also demonstrated a potential benefit in reducing the need for repeat procedures. Overall, both PASCAL and MitraClip were effective in reducing MR and improving cardiac function, indicating their utility in clinical practice.

In terms of procedure time, our meta-analysis did not find a significant difference between the PASCAL and MitraClip systems. Although early studies on PASCAL reported relatively longer procedure times, this may be attributed to less familiarity with the new device at that time [10]. However, Brath et al. (2021) reported a significant difference in procedure time, favoring PASCAL over MitraClip (72 ± 34 min. vs 131 ± 84 min., p = 0.007) [25]. This difference in procedure time could be explained by the fact that only one PASCAL device was implanted in 69% of patients to achieve significant reduction in MR, while 65% of patients received two or more MitraClip devices.

In terms of the device's safety, our meta-analysis found no significant difference between both systems for TEER. While PASCAL and MitraClip have comparable safety profiles, PASCAL may offer advantages for sufficient MR reduction and mitigate the risk of increased transmitral pressure gradient at discharge.

### Limitations

Our study has several limitations that should be acknowledged. First, most of the included studies were observational cohorts rather than RCTs, limiting the strength of our conclusions. Additionally, the relatively small number of studies (fewer than 10) hindered our ability to generate a funnel plot to assess publication bias. The follow-up periods were also relatively short, potentially limiting insights into the long-term effects of the interventions. A meta-regression analysis to identify which patients might benefit more from each technology would have strengthened the study but was not feasible due to the limited number of studies. Moreover, most studies did not distinguish between primary and secondary MR, preventing us from analyzing the interaction between device design and leaflet mechanics. The experience level with the MitraClip system may have introduced bias, given its longer availability compared to the PASCAL device; however, this also highlights the promising performance of the PASCAL system. Our study did not include the recently FDA-approved fourth-generation MitraClip, which could have different efficacy outcomes. Lastly, given that PASCAL is a newer device with less follow-up data than MitraClip, more randomized trials focusing on complex patient subgroups would provide further insights, though conducting additional trials might be challenging due to MitraClip established role.

### Future directions

Comparative studies between TEER devices, such as MitraClip and PASCAL, and emerging therapies, including transcatheter mitral valve replacement (TMVR) and Cardioband, are should be the future interesting directions for mitral valve therapy. As advancements in mitral valve interventions continue, future meta-analyses should integrate these novel treatments to ensure alignment with the evolving landscape of mitral valve therapy.

## Conclusion

Both the MitraClip and PASCAL systems are safe and effective for treating mitral regurgitation (MR), with comparable procedural success rates and safety profiles. However, the analysis did not show a statistically significant difference in MR severity reduction between the two systems, with similar results observed for both two-grade and one-grade reductions in MR. Additionally, there was no significant difference in patients with no improvement between the two devices. These findings suggest that while both devices are effective in reducing MR severity, further robust randomized controlled trials (RCTs) with longer follow-up are needed to confirm these results and explore the long-term outcomes and effectiveness of these TEER technologies.

## Supplementary Information

Additional file 1.

Additional file 2.

Additional file 3.

Additional file 4.

Additional file 5.

## Data Availability

No datasets were generated or analysed during the current study.

## Abbreviations

MR: Mitral Regurgitation
TEER: Transcatheter Edge-to-Edge Repair
MD: Mean Difference
RR: Risk ratio
CI: Confidence Interval
PRISMA: Preferred Reporting Items for Systematic Reviews and Meta-Analyses
RCTs: Randomized Clinical Trials
NYHA: New York Heart Association
EROA: Effective Regurgitant Orifice Area
