# Supplementary material for: Meta-analysis of MitraClip and PASCAL for transcatheter mitral edge-to-edge repair
Source: J Cardiothorac Surg. 2025 Jan 3;20:3. doi: 10.1186/s13019-024-03218-4 (PMC11697868; doi:10.1186/s13019-024-03218-4)
Supplement: Supplementary file 1 — Additional file 1. [file 13019_2024_3218_MOESM1_ESM.docx]

**Search strategy:** (Mitral) AND (Insufficiency OR Incompetence OR Regurgitation) AND (MitraClip) AND (Pascal)

**PubMed**

***Strategy:*** (Mitral) AND (Insufficiency OR Incompetence OR Regurgitation) AND (MitraClip) AND (Pascal)

**Results: 29**

**WOS**

***Strategy:*** (Mitral) AND (Insufficiency OR Incompetence OR Regurgitation) AND (MitraClip) AND (Pascal) (All Fields)

**Results: 37**

**Scopus**

***Strategy:*** (Mitral) AND (Insufficiency OR Incompetence OR Regurgitation) AND (MitraClip) AND (Pascal)

**Results: 85**

**Medline (Ovid)**

***Strategy:*** (Mitral) AND (Insufficiency OR Incompetence OR Regurgitation) AND (MitraClip) AND (Pascal)

**Results: 42**

**Cochrane**

***Strategy:*** Mitral) AND (Insufficiency OR Incompetence OR Regurgitation) AND (MitraClip) AND (Pascal)

**Results: 4**
