# Supplementary material for: Meta-analysis of MitraClip and PASCAL for transcatheter mitral edge-to-edge repair
Source: J Cardiothorac Surg. 2025 Jan 3;20:3. doi: 10.1186/s13019-024-03218-4 (PMC11697868; doi:10.1186/s13019-024-03218-4)
Supplement: Supplementary file 2 — Additional file 2. [file 13019_2024_3218_MOESM2_ESM.docx]

|  | **Supplementary Table .** Quality assessment criteria used for case-control studies through a modified version of Newcastle-Ottawa Scale for case-control studies. | | | | | | | | |
| --- | --- | --- | --- | --- | --- | --- | --- | --- | --- |
| **Study ID** | | **Barth et al. 2021** | **Errthum et al. 2021** | **Geis et al. 2022** | **Gerçek et al. 2021** | **Mauri et al. 2022** | **Schneider et al. 2022** | **Haschemi et al. 2022** |  |
| Sample selection criteria (****) | | **** | **** | **** | **** | **** | **** | **** |  |
| **1) Representativeness of the exposed cohort** (a) Truly representative (one star) (b) Somewhat representative (one star) (c) Selected group (d) No description of the derivation of the cohort | | a | a | a | a | a | a | a |  |
| **2) Selection of the non-exposed cohort** (a) Drawn from the same community as the exposed cohort (one star) (b) Drawn from a different source (c) No description of the derivation of the non exposed cohort | | a | a | a | a | a | a | a |  |
| **3) Ascertainment of exposure** (a) Secure record (e.g., surgical record) (one star) (b) Structured interview (one star) (c) Written self report (d) No description (e) Other | | a | a | a | a | a | a | a |  |
| **4) Demonstration that outcome of interest was not present at start of study** (a) Yes (one star) (b) No | | Yes | Yes | Yes | Yes | Yes | Yes | Yes |  |
| Comparability (**) | | ** |  | ** | * | ** | ** | ** |  |
| **1) Comparability of cohorts on the basis of the design or analysis controlled for confounders** (a) The study controls for age, sex and marital status (one star) (b) Study controls for other factors (list) (one star) (c) Cohorts are not comparable on the basis of the design or analysis controlled for confounders | | a | c | a | b | a | a | a |  |
| Exposure (***) | | *** | ** | *** | *** | *** | *** | *** |  |
| **1) Assessment of outcome** (a) Independent blind assessment (one star) (b) Record linkage (one star) (c) Self report (d) No description (e) Other | | b | c | b | b | b | b | b |  |
| **2) Was follow-up long enough for outcomes to occur** (a) Yes (one star) (b) No | | a | a | a | a | a | a | a |  |
| **3) Adequacy of follow-up of cohorts** (a) Complete follow up- all subject accounted for (one star) (b) Subjects lost to follow up unlikely to introduce bias- number lost less than or equal to 20% or description of those lost suggested no different from those followed. (one star) (c) Follow up rate less than 80% and no description of those lost (d) No statement | | a | a | a | a | a | a | a |  |
| **Summary quality score (Overall risk of bias)** | | **Low** | **Moderate** | **Low** | **Low** | **Low** | **Low** | **Low** |  |
| ID, identification; Score, * = 1, no star (*) = 0. NOS total score: 0 to 3; High-risk of bias, 4 to 6; Moderate-risk of bias, ≥ 7; Low-risk of bias  The maximum score of each item is represented in parentheses. | | | | | | | | | |
