# Supplementary material for: Meta-analysis of MitraClip and PASCAL for transcatheter mitral edge-to-edge repair
Source: J Cardiothorac Surg. 2025 Jan 3;20:3. doi: 10.1186/s13019-024-03218-4 (PMC11697868; doi:10.1186/s13019-024-03218-4)

**Supplementary 4**

**Supplementary Figure S1.** Forest plot of mean difference (MD) and 95% confidence interval (CI) in procedure time.


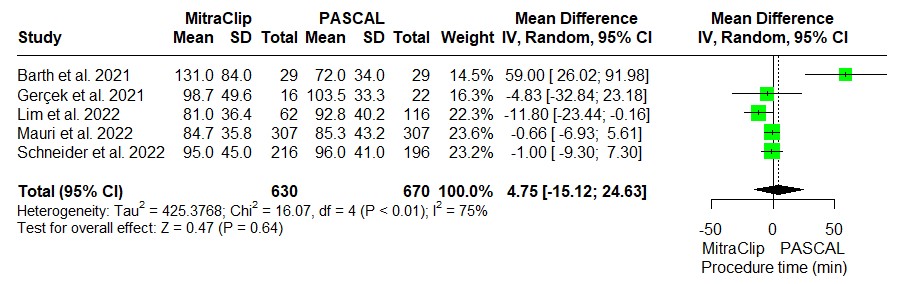


**Supplementary Figure S2.** Sensitivity analysis leave one-out test in the outcome of procedure time.


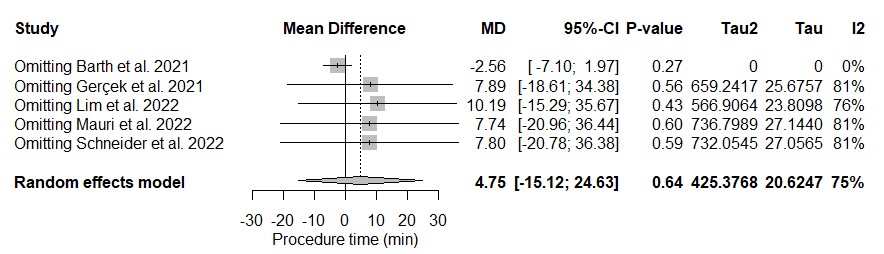


**Supplementary Figure S3.** Forest plot of risk ratio (RR) and 95% confidence interval (CI) in procedural success.


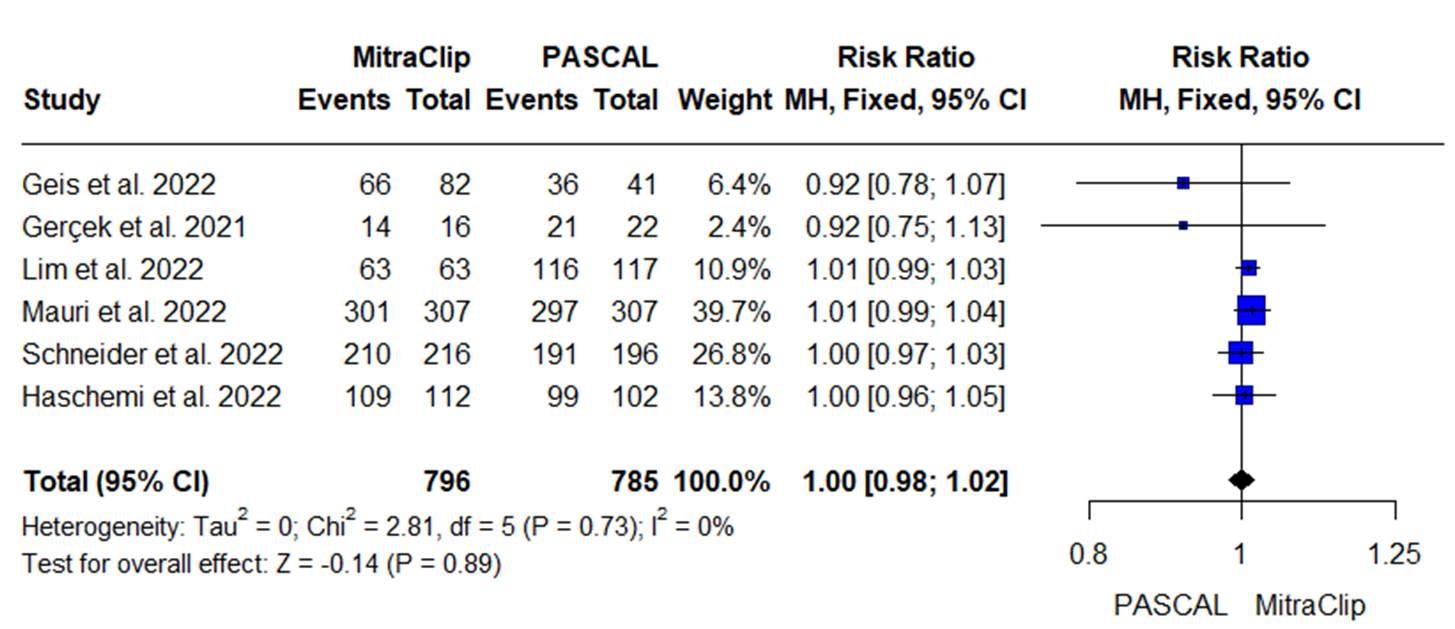


**Supplementary Figure S4.** Forest plot of risk ratio (RR) and 95% confidence interval (CI) in device success.


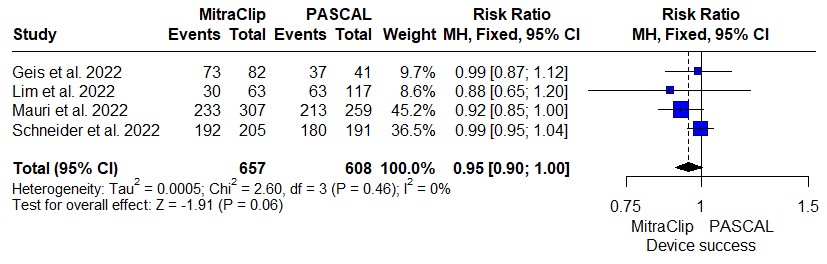


**Supplementary Figure S5.** Forest plot of risk ratio (RR) and 95% confidence interval (CI) in major bleeding.


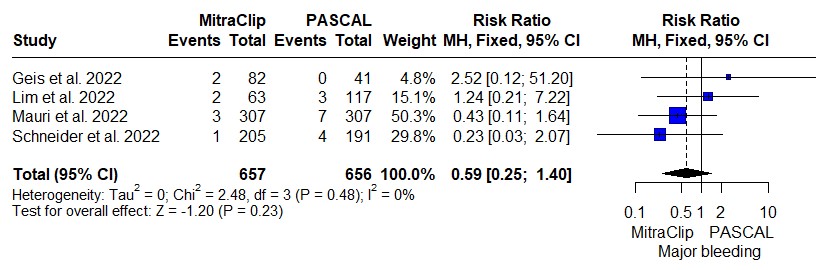


**Supplementary Figure S6.** Forest plot of risk ratio (RR) and 95% confidence interval (CI) in reintervention.


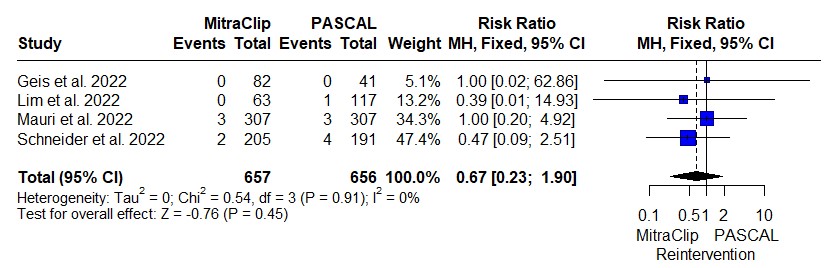


**Supplementary Figure S7.** Forest plot of risk ratio (RR) and 95% confidence interval (CI) in all-cause mortality.


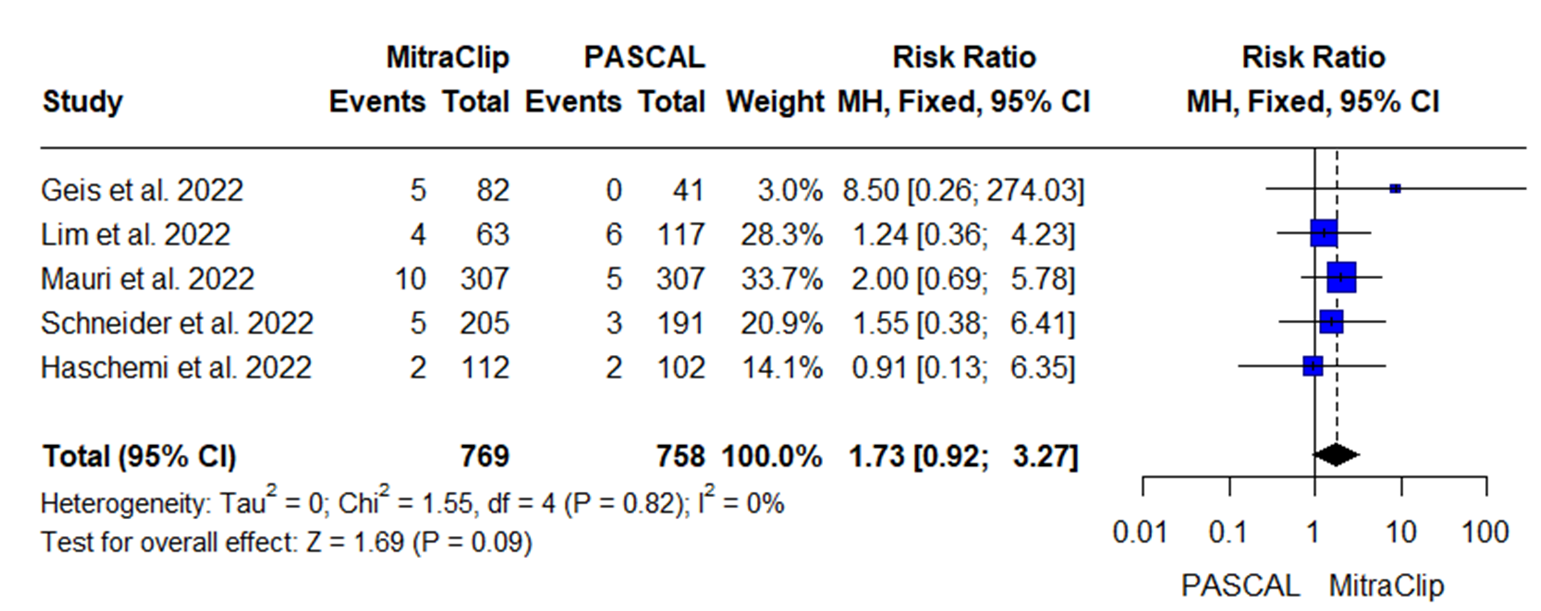

Supplement: Supplementary file 4 — Additional file 4. [file 13019_2024_3218_MOESM4_ESM.docx]
