# Supplementary material for: Meta-analysis of MitraClip and PASCAL for transcatheter mitral edge-to-edge repair
Source: J Cardiothorac Surg. 2025 Jan 3;20:3. doi: 10.1186/s13019-024-03218-4 (PMC11697868; doi:10.1186/s13019-024-03218-4)
Supplement: Supplementary file 5 — Additional file 5. [file 13019_2024_3218_MOESM5_ESM.docx]

**Supplementary materials**

- **Supplementary 1.** Search Strategy for each database.
- **Supplementary 2.** Quality assessment criteria used for case-control studies through a modified version of the Newcastle-Ottawa Scale for case-control studies.
- **Supplementary 3.** Cochrane risk of bias 2 for RCT quality assessment.
- **Supplementary 4.** (Figure S1 – S
  - **Figure S1.** Forest plot of mean difference (MD) and 95% confidence interval (CI) in procedure time.
  - **Figure S2.** Sensitivity analysis leave one-out test in the outcome of procedure time.
  - **Figure S3.** Forest plot of risk ratio (RR) and 95% confidence interval (CI) in procedural success.
  - **Figure S4.** Forest plot of risk ratio (RR) and 95% confidence interval (CI) in device success.
  - **Figure S5.** Forest plot of risk ratio (RR) and 95% confidence interval (CI) in major bleeding.
  - **Figure S6.** Forest plot of risk ratio (RR) and 95% confidence interval (CI) in reintervention.
  - **Figure S7.** Forest plot of risk ratio (RR) and 95% confidence interval (CI) in all-cause mortality.
